# Improving Accuracy of Malaria Diagnosis in Underserved Rural and Remote Endemic Areas of Sub-Saharan Africa: A Call to Develop Multiplexing Rapid Diagnostic Tests

**DOI:** 10.1155/2020/3901409

**Published:** 2020-02-21

**Authors:** Rasheed O. Makanjuola, Andrew W. Taylor-Robinson

**Affiliations:** ^1^Department of Biology and Biotechnology, University of Pavia, Lombardy, Italy; ^2^Department of Microbiology, Edo University, Iyamho, Edo State, Nigeria; ^3^Infectious Diseases Research Group, School of Health, Medical & Applied Sciences, Central Queensland University, Brisbane, Australia

## Abstract

Clinical infection with malaria, caused by parasites of the genus *Plasmodium*, is considered a serious medical condition with the potential to become a life-threatening emergency. This is especially relevant to low-income countries in tropical and subtropical regions of the world where high rates of malaria-related morbidity and mortality are recorded. As a means to combat this major global public health threat, rapid and effective diagnosis remains the frontline action to initiate a timely and appropriate medical intervention. From all the approaches to parasite detection, rapid diagnostic tests, so-called RDTs, are the easiest to use and most cost-effective. However, some of the limitations inherent in this methodology could hinder effective patient treatment. A primary drawback is that the vast majority of commercially available RDTs detect only one of the five species of human malaria, *P. falciparum*. While this is the main cause of infection in many areas, it excludes the possibility of infection with another parasite (*P. vivax, P. ovale, P. malariae*, and *P. knowlesi*) or of mixed infections containing different species. Hence, a diagnosis of non-*P. falciparum* malaria is missed. In turn, in resource-constrained settings where optimal microscopy is not available, a misdiagnosis of bacterial infection based on signs and symptoms alone often results in an inappropriate prescription of antibiotics. Here, we discuss how effective diagnosis of malaria and indiscriminate use of antibiotics in sub-Saharan Africa, a hot spot for *P. falciparum* transmission, may both be addressed by the development of innovative multiplexing RDTs that detect two or more species of *Plasmodium*.

## 1. Introduction

Malaria is a potentially life-threatening mosquito-transmitted infectious disease that is of worldwide public health concern. Infection in humans is caused by each of five species of blood-dwelling protozoan parasites belonging to the genus *Plasmodium*, namely, *P. falciparum, P. vivax, P. ovale, P. malariae*, and *P. knowlesi*. Infective, motile stages of the parasite, sporozoites, are transferred between humans through the bite of an infectious female *Anopheles spp.* mosquito that serves as an intermediary vector of transmission [1]. Sporozoites home to the liver, where they undergo an immunologically quiescent intrahepatocytic multiplication phase for 7–10 days before the resultant merozoites are released into the peripheral blood. The asexual erythrocytic cycle that follows, repeated every 24–72 hours in a species-dependent manner, is associated with the pathogenesis of the disease [2].

The consequences of *Plasmodium* infection vary in severity depending on the species and on host factors, including the level of a person's immunity, which is correlated with their history of parasite exposure [3, 4]. By convention, malaria is classified as asymptomatic (when a person harbours the parasite but shows no obvious signs of illness), uncomplicated, and severe (associated with complicated manifestations) [5]. Initial symptoms are characterized by a low-grade fever, rigors, and myalgia. Paroxysmal symptoms can occur suddenly, in synchrony with the haemolysis of parasitized erythrocytes, and progress to drenching sweats, high fever, and exhaustion. Severe malaria is frequently fatal; it presents as severe anaemia and multiorgan dysfunction, including cerebral and renal manifestations [2].

According to the World Health Organization (WHO) report of 2018 on malaria, in the preceding year, 219 million cases of symptomatic infection were reported from 97 nations and territories, of which 435,000 were fatal [6]. Sub-Saharan Africa contributed significantly to the global burden of malaria infection (92%). In fact, four countries in this region accounted for nearly half of total cases: Nigeria (25%), Democratic Republic of Congo (11%), Mozambique (5%), and Uganda (4%) [6]. Following a period of unprecedented success in global malaria control from the turn of this century driven by the Roll Back Malaria initiative, since 2015 progress has stalled. At present, more than 3 billion people are still at risk of infection. With a view to addressing this alarming situation, the WHO has set new goals for malaria reduction, including the fact that by the year 2030 there should be a reduction of global malaria incidence and mortality rates of at least 90%, as well as the elimination of the disease in at least 35 currently endemic countries [7]. In order to attain these ambitious targets, the strategies prioritized by the WHO were universal access to malaria prevention, drugs and diagnosis, elimination, and surveillance [8, 9].

## 2. Accurate Diagnosis Informs Effective Treatment

A rapid and effective diagnosis remains the best prospect for a quick therapeutic response to malaria infection. Yet, despite the fact that considerable advances have been made in the design of novel diagnostic approaches, none of these has so far fulfilled its initial promise in terms of disease management and control in malaria-endemic environments [10]. Most of the available rapid malaria diagnostic kits target *P. falciparum* only [11] and so will help to alleviate the burden of the causative agent of the most serious human infection. In our opinion, however, focusing exclusively on this one species is unlikely to achieve extensive and sustained success. In support of this assertion are findings from malaria epidemiological surveillance research that indicate the extensive overlap of geographical distribution between *Plasmodium* species, reflective of the range of *Anopheles* mosquito vectors that transmit these parasites, in parts of sub-Saharan Africa [12]. As a consequence, an individual may become infected with, and harbour, separate species on different occasions or even at the same time.

The extent of exposure to multiple parasites is exemplified by a study based on the traditional, accurate, but slow method of optical microscopical examination of peripheral blood smears that was performed on patients from seven primary healthcare centres in Ogun state, Nigeria [13]. Of 384 samples, 273 (71.1%) tested positive for any malaria parasite but four species were detected: *P. falciparum*, *P. malariae*, *P. ovale*, and *P. vivax* accounted for 95.6%, 3.3%, 0.7%, and 0.4% of cases, respectively [13]. Using this finding as a guideline from which to extrapolate, the 4.4% infection rate caused by malaria parasites other than *P. falciparum* is extremely significant in Nigeria, a country where over 200 million people live in areas that expose them to malaria transmission. These non-falciparum species are of considerable clinical importance; for example, *P. vivax* and *P. ovale* can form latent intrahepatic hypnozoites that may cause disease several months or even years after the primary infection [14, 15]. Diagnosis of systemic disease caused by *P. malariae* a substantial time after a person has left a malaria-endemic region has been recorded [16]. Incidences of parasitaemia recrudescence, drug treatment failure, and even drug resistance attributable to misdiagnosis of primary infections caused by non-falciparum parasites or as species coinfecting with *P. falciparum* have been reported [17–19].

In this brief review, we outline existing methods for the detection of blood stage *Plasmodium* and discuss their limitations in the diagnosis of malaria infection (Table 1). This highlights the need to introduce a new generation of rapid diagnostic tests (RDTs) that are faster and cheaper and have multiplexing functionality for the detection of more than one *Plasmodium* species. Moreover, this diagnostic advance would also help to reduce the widespread misuse of antibiotics in malaria-endemic developing nations [20]. This often follows an incorrect predictive diagnosis of a nonmalarial illness that is predicated solely on a person's nonspecific febrile symptoms at first presentation [21].

## 3. Symptom-Based Diagnosis

Malaria is the most common cause of fever in endemic environments, so it is understandable that it is the diagnosis that is considered as the default option if a person presents with a fever of unknown origin. Inevitably, this mindset leads to overdiagnosis of *P. falciparum* [22]. However, as malaria shares clinical signs and symptoms that are a feature common to a range of other febrile illnesses, a presumption of malaria infection is not fail-safe [21]. There is often a reliance on this traditional, observational diagnostic approach in remote resource-limited settings. Its degree of accuracy depends on the transmission intensity of malaria, the prevalent species of *Plasmodium* parasite, and other prevailing causes of fever in any given region. These factors vary from one location to another, may be unstable, and thus are not completely reliable [5, 23].

It can be reasonably assumed that, in primary healthcare settings in sub-Saharan Africa and other regions where this diagnostic rationale is prevalent, both antimalarial and antibiotics drugs are administered to patients [20–22]. It is likely that this practice contributes significantly to the ongoing public health problem of antibiotics abuse and thereby elevates the risk of the spread of antimicrobial resistance genes among pathogenic bacteria [20]. Therefore, in order to realize an appropriate medical response, it is strongly advisable to follow a predictive clinical or self-diagnosis of this kind with confirmatory diagnostic tests.

## 4. Microscopy-Based Diagnosis

This is the longest established laboratory-based malaria diagnostic technique and involves direct visualization of asexual intraerythrocytic stages of the *Plasmodium* parasite. The conventional procedure involves preparing a smear (film) of peripheral blood on a glass microscope slide followed by fixing with absolute methanol, staining in Giemsa solution, and visualizing by optical microscopy [24]. Giemsa is composed of eosin and methylene blue (azure). Eosin stains the parasite nucleus red while methylene blue stains the cytoplasm blue. This method reveals quantitative and qualitative diagnostic information in terms of the infecting *Plasmodium* species and the proportion of infected erythrocytes (parasite density; parasitaemia), typically from the examination of thick and thin blood smears, respectively [25]. Moreover, the exact stage of the asexual erythrocytic cycle at which a blood sample was taken may also be determined, which is useful to match with that patient's clinical manifestations. If no parasites are present at first screening, the test may be repeated every 8 hours for up to 48 hours if malaria is suspected. When performed by a trained malaria microscopist, the degree of accuracy of both detection and identification is high. Consequently, the microscopic examination of blood smears is the easiest and most reliable test for malaria diagnosis and thus is firmly established as the ‘gold standard' method [10]. However, it is not without limitations; for instance, it has a questionable sensitivity at low-level parasitaemia (≤50 parasitized erythrocytes/*μ*l blood). Furthermore, due to inadequate facilities and/or lack of equipment and reliable electricity supply, combined with a shortage of experienced personnel, this approach becomes inaccessible in many primary healthcare centres in remote communities, locations where malaria may be extremely endemic [9, 25] (Table 1).

## 5. Molecular-Based Diagnosis

The molecular-based diagnostic approach is a highly sensitive and specific method to determine the molecular signature of infectious agents through nucleic acid amplification. Interestingly, this technique allows the identification of the *Plasmodium* parasite to species level [26] and, unlike the microscopy method, also detects low-level parasitaemia [27]. The assays include those based on conventional polymerase chain reaction (PCR), nested PCR, real-time PCR, droplet digital PCR, and isothermal amplification. Unfortunately, the thermal cycler, or ‘PCR machine', is expensive to purchase and to maintain, while the cost of reagents per test is prohibitive in low-income settings. Furthermore, its operation and data analysis require highly trained personnel (Table 1). These factors combine to make a diagnosis by this means all but inaccessible in economically ailing countries in which malaria is endemic. In addition, even if there is the option to transport samples from remote locations to regional laboratories for processing, the results of molecular diagnoses are not often available quickly enough to influence medical intervention [28]. While these assorted sensitive molecular assays may not be of direct therapeutic benefit under field conditions, they find applied value as a research tool when absolute quantification of very low parasite density is required or when mixed infections need identification [26]. This may enable detailed insights into the epidemiology of each *Plasmodium* species in various transmission settings, notably to follow subpatent infection of individuals who act as asymptomatic reservoirs in areas targeting malaria elimination.

## 6. Immunology-Based Diagnosis

As a diagnostic approach, immunodiagnosis is characterized by exploiting an antigen-antibody reaction as the primary means of detection [29]. Unfortunately, while providing useful information in regard to a person's history of exposure to *Plasmodium* parasites, this serological method cannot differentiate between past and present malaria infections. Therefore, this method is intrinsically less suited to the rapid detection of suspected current infection in order to inform effective treatment and more beneficial to malaria epidemiological studies and surveillance programs [23] (Table 1). For instance, competitive enzyme-linked immunosorbent assay (ELISA) can be used as a diagnostic tool to determine in a particular malaria-endemic study site the species specificity of IgG antibody responses, and epidemiology of exposure, to merozoite surface protein-1 (MSP-1), an immunodominant asexual stage *Plasmodium* antigen [30]. Serological responses generally increase with exposure, and thus, as an indirect correlate, with age, in regions of stable malaria transmission. This epidemiological pattern reflects the acquisition of protective immunity that develops after repeated exposure [31]. However, the age dynamics associated with serological responses to malaria are not apparent in seasonal and unstable malaria transmission areas such as eastern Sudan [32], where malaria affects all age groups.

## 7. Rapid Diagnostic Tests (RDTs)

In contrast to immunodiagnosis, the malaria RDT is based on an immunochromatographic technique and is associated with the detection of *Plasmodium*-specific antigens in blood samples using monoclonal antibodies impregnated on a nitrocellulose membrane [33]. First developed in the 1990s [34], RDTs are fast becoming the most used method to test for malaria diagnosis among suspected malaria patients in public health facilities. According to the latest available figures [6], an estimated 75% of malaria tests were conducted using RDTs in 2017, up from 40% in 2010. There are more than 200 different hand-held RDT kits that are currently commercially available [35], the great majority of which have specificity for *P. falciparum* histidine-rich protein 2 (*Pf* HRP-2) and are thus limited to detecting malaria caused by *P. falciparum* [36]. Other asexual stage antigens available to tests, such as *Plasmodium* lactate dehydrogenase or aldolase enzymes, are only genus-specific [11, 25]. In sub-Saharan Africa and other zones where *P. falciparum* predominates, the present WHO recommendation is to use RDTs that target *Pf* HRP-2. Hence, two-thirds of the 276 million tests supplied to national malaria programs globally in 2017 detected *P. falciparum* only [6].

Notwithstanding the fact that most of the present generation of RDTs satisfy the WHO's guideline ASSURED (Affordable, Sensitive, Specific, User-friendly, Rapid and Robust, Equipment-free, and Deliverable to end-users) criteria for appropriate diagnostic assays [9, 37], there exist several restraints on their suitability for malaria diagnosis. These include their inability to quantify parasite density, false-positive results due to persistence of *Pf* HRP-2 in the blood sample for several days after clearance of infection, false-negative results due to mutation (deletion) in the gene encoding *Pf* HRP-2 protein [25, 36], and a high sensitivity threshold of approximately 200 parasitized erythrocytes/*µ*l blood [23] (Table 1). Despite all these constraints, RDTs are increasingly indispensable tools for point-of-care diagnosis of malaria infection in both remote and urban healthcare centres. However, their utility in settings of declining transmission intensity, where malaria elimination is the goal, has been called into question [38, 39].

A major advantage of RDTs over other ways of diagnosing malaria (Table 1) is their relative affordability, thus complying in this important regard with WHO guidelines [23], which called for a change from presumptive to test-based treatment. In a recent study of Nigerian pharmacies, the average cost of an RDT kit and that of the pharmacist's time spent in administering the test were calculated to be US$ 0.15 and US$ 0.41, respectively [40]. More than 82% of pharmacy patients who formed the survey respondents preferred to take an RDT before treatment, on average willing to pay US$ 1.23 per diagnosis. In a country where malaria accounts for around 60% of outpatient visits and 30% of hospitalizations [41], this provided a benefit-cost ratio of 6.7, indicating that malaria treatments based on diagnosis by RDT are extremely cost-beneficial. The reliance on the private health sector in Nigeria is broadly representative of most sub-Saharan countries, in which significant proportions of the populace engage nonmedical practitioners, particularly licensed community pharmacists and less tightly regulated medicine vendors, as their default point of care for febrile illnesses [42, 43].

## 8. Discussion

Poor diagnosis of infection is a causal factor in the continuing major economic burden that malaria places on low-income countries with higher malaria incidence [44]. Accurate, rapid, and cost-effective diagnosis is one of the strategies highlighted by the WHO towards the control of malaria. Although most of the existing diagnostic approaches have merit, unfortunately, each has a number of limitations. Among these are microscopy and molecular-based diagnosis. Despite the fact that these two techniques are the preferred option in terms of accuracy, sensitivity, and species-specific detection, they are not available in hard-to-reach remote areas where malaria is endemic. The principal reasons for this are that in both cases they require well-trained personnel and the equipment is expensive. Thus, there is a pressing requirement for diagnostic tools with the realistic prospect of regional point-of-care applications [45]. Moreover, ideally, these should move away from a one-disease/one-test philosophy that does not meet the complex health needs of populations in resource-constrained settings where coinfections are common [46].

As a consequence of the aforementioned drawbacks of alternative diagnostic techniques, being easier to operate, inexpensive, and cost-effective, RDTs remain the only valid option to support primary healthcare provision in underserved communities in impoverished remote areas [47]. However, to our knowledge, all of the currently manufactured RDTs have single specificity, with availability in sub-Saharan Africa almost exclusively restricted to those for *P. falciparum* [35]. Across this region, therefore, since different *Plasmodium* species are not included in the test, any malaria infection caused by a species other than *P. falciparum* that provides a negative result may not be considered when making a diagnosis. In these circumstances, it is highly likely that the febrile illness will be presumed to be of bacterial or viral origin. Hence, the patient will not receive malaria-appropriate therapy, the nondiagnosis of which jeopardizes their health outcome. There is, therefore, a need to focus on improving the care of RDT-negative people with fever living in malaria-endemic settings [48]. More generally, inappropriate treatment of malaria with antibiotics, prescribed by qualified clinicians and nonmedical practitioners alike acting on an uninformed diagnosis, contributes to their continuing global misuse [20]. In turn, this action escalates the global public health crisis surrounding antimicrobial resistance [49].

Up till now, a multiplex RDT for the simultaneous detection of more than one species of *Plasmodium* has not been brought to the market. However, the anticipation that it may be possible in the future is heightened from the recently developed and tested multiplex application of two other diagnostic techniques founded on nucleic acid amplification to identify species of *Plasmodium* [49, 50]. For the first, a real-time PCR for detecting human malaria in the nonendemic setting of the Netherlands was used in a routine diagnostic laboratory to screen blood samples provided by travelers and migrants from malaria-endemic regions [50]. This showed strong agreement with microscopy, demonstrated higher sensitivity and improved specificity, but took much longer to deliver results. For the second, a paper-coated, DNA-based assay was developed for high-quality, fast precision malaria diagnostics for trial in rural Uganda [51]. The test enabled the diagnosis of malaria species in patients from a finger prick of whole blood and was both highly sensitive and specific, detecting either *P. falciparum* or pan-*Plasmodium* malaria in 98% of infected individuals in a double-blind first-in-human study [51]. This advance provides a proof of principle for in-field use of a diagnostic test that performs similarly to the laboratory-based real-time PCR test [49]. While it detects low parasite densities, as with the real-time PCR, it is not yet feasible to roll out at a low cost for widespread use. This highlights the logistical and economic challenges associated with developing and implementing new diagnostic technologies in the field, without access to complex instrumentation, centralized laboratories, or infrastructure.

## 9. Conclusion

Given the present scenario of suboptimal malaria diagnosis, we advocate for novel multiplexing RDTs that can diagnose two or more *Plasmodium* species simultaneously, dependent upon their environmental prevalence. It is envisaged that this key diagnostic development will make a major contribution to addressing cost-effective detection of malaria infection caused by non-*P. falciparum*. This will directly benefit point-of-care testing and thereby better inform treatment in rural and remote resource-limited settings in sub-Saharan Africa. As a knock-on effect, improved malaria diagnosis through the introduction of multiplexing RDTs should reduce indiscriminate use of antibiotics for the treatment of febrile illnesses in this and other malaria-endemic regions.

## Figures and Tables

**Table 1:** Advantages and disadvantages of various malaria diagnostic tests.

Malaria diagnostic test	Advantages	Disadvantages
1. Microscopy-based	(i) Very accurate and specific	(i) Requires highly trained personnel
	(ii) Provides both qualitative (*Plasmodium* species) and quantitative (parasite density) data	(ii) Requires reliable electricity supply
	(iii) Reveals the stage of malaria infection	(iii) Low sensitivity

2. Molecular-based	(i) Highly sensitive and specific	(i) Requires highly trained personnel
	(ii) Distinguishes between different *Plasmodium* species	(ii) Very expensive equipment
		(iii) Time-consuming

3. Immunology-based	(i) Provides information on malaria epidemiological surveillance	(i) Cannot differentiate between past and present infections

4. Rapid diagnostic test (RDT)	(i) Provides results very quickly	(i) Unable to quantify parasite density
	(ii) Inexpensive	(ii) Cannot differentiate between past and present infections
	(iii) Very easy to use with little training	(iii) Mutation in the gene encoding the antigen can affect the result
